# iTRAQ-based proteomic analysis of sperm reveals candidate proteins that affect the quality of spermatozoa from boars on plateaus

**DOI:** 10.1186/s12953-021-00177-9

**Published:** 2021-07-30

**Authors:** Yanling Zhao, Yaomei Wang, Feipeng Guo, Bo Lu, Jiale Sun, Jianzhou Wang, Zili Ren

**Affiliations:** College of Animal Science, Tibet Agricultural and Animal Husbandry University, Linzhi, Tibet 860000 P.R. China

**Keywords:** Boar, Comparative proteomics, iTRAQ, Sperm quality, Plateaus

## Abstract

**Background:**

Tibetan pigs (TP) exhibit heritable adaptations to their hypoxic environments as a result of natural selection. However, candidate proteins that affect the sperm quality of boars on plateaus have not yet been clearly investigated.

**Methods:**

In this study, to reveal the candidate proteins that affect the quality of spermatozoa of boars on plateaus, we analyzed the sperm quality using computer-assisted semen analysis (CASA) system and reactive oxygen species (ROS) levels. We also compared the proteomes of sperm proteomes between TP and Yorkshire pigs (YP) raised at high altitudes using the isobaric tags for relative and absolute quantitation (iTRAQ) in combination with the liquid chromatography-tandem mass spectrometry (LC–MS/MS) proteomic method, and confirmed the relative expression levels of the four proteins by western blotting.

**Results:**

The sperm quality of the TP was superior to that of the YP on plateaus. Of the 1,555 quantified proteins, 318 differentially expressed proteins (DEPs) were identified. Gene ontology (GO) analysis revealed that the DEPs were predominantly associated with the sorbitol metabolic process, removal of superoxide radicals, cellular response to superoxide, response to superoxide and regulation of the mitotic spindle assembly. The Kyoto Encyclopedia of Genes and Genomes (KEGG) pathways were mainly enriched in pathways involved in the regulation of the actin cytoskeleton, glutathione metabolism, oxidative phosphorylation, and estrogen signaling. Based on the protein–protein interaction (PPI) network analysis, we identified 8 candidate proteins (FN1, EGF, HSP90B1, CFL1, GPX4, NDUFA6, VDAC2, and CP) that might play important roles and affect the sperm quality of boars on plateaus. Moreover, the relative expression levels of four proteins (CFL1, EGF, FN1, and GPX4) were confirmed by western blot analysis.

**Conclusions:**

Our study revealed 8 candidate proteins (FN1, EGF, HSP90B1, CFL1, GPX4, NDUFA6, VDAC2, and CP) that affect the sperm quality of boar on plateaus and provide a reference for further studies on improving sperm quality and the molecular breeding of boars on plateaus.

**Supplementary Information:**

The online version contains supplementary material available at 10.1186/s12953-021-00177-9.

## Background

On plateaus, the reproductive system and antioxidants involved in oxidative stress in male boars are affected by hypobaric hypoxia [1]. Tibetan pigs (TP) is a unique and geographically isolated pig breed that inhabits high-altitude regions of the Qinghai-Tibetan Plateau and exhibits heritable adaptations to its hypoxic environment as a result of natural selection [2]. Previously, we determined that the sperm motility of TP in low-altitude and high-altitude areas was 0.84 and 0.82, respectively (no significant difference) [3]. But we have determined that the sperm motility of Yorkshire pigs (YP) that migrated from the lowlands to the plateaus, was 0.80 and 0.73 respectively (a significant decrease). That is, the sperm motility of TP is higher than that of YP on plateaus, but analyses of other sperm quality parameters of TP and YP raised at high altitudes have not yet been conducted. Reactive oxygen species (ROS) have been recognized to negatively affect sperm survival and fertility when their production exceeds the formation and peroxidation of membrane lipids, and the reduction of sperm motility may be due to ROS-induced loss of ATP utilization or lesions in the contractile apparatus of the flagellum [4]. Therefore, it is necessary to evaluate sperm ROS levels when performing sperm quality analysis.

Multiple genes and biological pathways control sperm quality, which is the most economically important trait of sperm. Proteomic data enable a better understanding of sperm biochemistry [5]. In-depth proteomic analyses of boar spermatozoa have been performed using shotgun and gel-based methods [6]. The proteome of pig spermatozoa is remodeled during ejaculation [7]. The comparative proteomes of prenatal muscle tissues among TP, Wujin pigs and large White pigs have been analyzed using the isobaric tag for relative and absolute quantification (iTRAQ) [8]. Comparative proteomics of TP spermatozoa from high and low altitudes has been performed to elucidate the mechanisms underlying the high-altitude tolerance of TP spermatozoa [3]. However, the sperm proteomes of TP and YP raised at high altitudes have not yet been compared. iTRAQ is a powerful technique for quantitatively analyzing proteomes [9] and has high sensitivity, good repeatability, the ability to label almost all enzymatic peptides, and the ability to label 8 samples simultaneously [10].

In this study, to reveal candidate proteins that affect the sperm quality of boars on plateaus (YP was used as the control group), we analyzed sperm quality, compared the sperm proteomes of TP and YP raised at high altitudes using iTRAQ, and confirmed the relative expression levels of four identified proteins by western blotting. This study provides a reference for further studies investigating improvements of sperm quality and the molecular breeding of boars on plateaus.

## Methods

### Animal samples

Experiments were performed using pigs from two different populations: Tibetan pigs living in highlands (Linzhi, 3,000 m, TP) and Yorkshire pigs that migrated from lowlands (Beijing, 100 m) to highlands (Linzhi, 3,000 m, YP) approximately 3 yr ago. TP and YP were raised in the Tibet Agricultural and Animal Husbandry University Farm (Linzhi, 3,000 m) and Tibet Linzhi Ga Ma Breeding Co., Ltd. (Linzhi, 3,000 m), respectively, and ten boars (1.5 yr old with normal fertility and nutrition levels) from each population were used in this study (YP was used as the control group).

### Semen collection and quality analysis

Twenty fresh semen samples, one per boar, were obtained from 10 TP and 10 YP by using the gloved-hand technique. After semen collection, no adverse effects on the health and growth of pigs were observed. Sperm quality was measured by sperm motility parameters and ROS levels. A computer-assisted semen analysis (CASA) system (Hamilton Thorne Research, Beverly, MA, USA) was used to measure the sperm concentration, motility, and VAP according to the manufacturer's instructions. In short, semen was incubated at 37 °C for 10 min, and then, 3 μL of semen was dropped onto a preheated (37 °C) Makler sperm count board, and sperm motility and other parameters were assessed by using the CASA system. At least 3 visual fields were observed to obtain an average. Diff-Quik staining was performed to detect the abnormal sperm rate [11]. Briefly, semen was fixed in 3.7% paraformaldehyde for 10 min, washed with PBS two times, resuspended in PBS, smeared, and stained according to the manufacturer's protocol for the Diff-Quik Stain Kit (D030-1–2, Nanjing Jiancheng Bioengineering Institute, Nannjing, China). The experiments were repeated three times, and at least 200 spermatozoa were counted in each count at 400 × under and ordinary light microscope. According to the manufacturer's protocol for the ROS assay kit (S0033M, Shanghai Beyotime Biotechnology Co. Ltd, Shanghai, China), the sperm ROS levels were evaluated by using the probe 2′,7′-dichlorodihydrofluorescein diacetate (DCFH-DA). Briefly, the semen samples were washed with PBS three times, resuspended and incubated with 10 μM DCFH-DA at 37 °C in the dark for 25 min. For the determination of the deesterification of intracellular DCFH-DA to dichlorodihydrofluorescein via oxidization by ROS, which has strong fluorescence, the fluorescence intensity was monitored using a fluorescent microplate reader (Biotek Synergy, SynergyH4, USA) at an excitation wavelength of 488 nm and at an emission wavelength of 525 nm.

### Protein preparation

To remove seminal plasma and contamination (e.g., extender components and somatic cells, such as leukocytes and testicular cells), the semen samples were centrifuged at 500 × g for 20 min in a discontinuous (70% [v/v] and 35% [v/v]) Percoll gradient (Sigma, St Louis, MO, USA), and then, the sperm pellets were washed 3 times with cold phosphate-buffered saline (PBS). For protein extraction, each sperm sample (3 × 10^8^ spermatozoa) was resuspended in lysis buffer (8 M urea, 4% CHAPS, 50 mM DTT and protease inhibitor, pH 8.0) at 4 °C. The lysates were centrifuged at 10,000 g for 30 min to remove insoluble material, and the supernatants were collected for further analysis. The protein content was measured with a Bradford protein assay kit (P0006C, Beyotime Institute of Biotechnology, Nanjing, China).

### Protein labeling and liquid chromatography coupled with tandem mass spectrometry (LC–MS/MS)

Equal aliquots of proteins (100 mg each) from the sperm samples were digested using filter-assisted sample preparation (FASP) as previously described [12]. The resulting TP and YP peptides were labeled 116 (TP1), 121 (TP2), 113 (YP1) and 119 (YP2) according to the instructions of the iTRAQ® Reagent-8PLEX Multiplex Kit (4,381,663, AB SCIEX, USA). The proteomic experiments required a more complex peptide mixture than the protein mixture. As a consequence, the peptides needed to be separated by two dimensional liquid chromatography. The first dimension was performed with a high-pH C18 reverse phase separation; 8–10 fractions were collected first, and each fraction was separated by a two-step nano-LC separation combined with high resolution mass spectrometry. The aim of the two dimmensional LC was to simplify the peptide component to increase the peptide signal for MS and allow more proteins to be identified. The combined peptide mixture was resuspended in buffer A (98% ddH_2_O, 2% acetonitrile, ACN, pH 10.0) and prefractionated by high-pH reverse-phase liquid chromatography (hp-RPLC) using an XBridge C18 column (130 Å, 3.5 µm, 4.6 mm × 250 mm,Waters, Milford, MA, USA) and an HPLC system (e2695, Waters, Milford, MA, USA) at a flow rate of 0.5 mL/min. The column was eluted with a 51 min gradient of 0 ~ 5% buffer B (98% acetonitrile, pH 10.0) for 5 min, 5 ~ 35% buffer B for 45 min, and 35 ~ 50% buffer B for 10 min at a flow rate of 1 mL/min. Forty fractions were collected and pooled into ten aliquots. The fractions were desalted using Zip-Tip C18 Tips (Millipore, USA; Cat. 87,782), suspended in buffer A (2% ACN, 0.1% FA), and analyzed on a nano-LC system (Easy nLC 1000, Thermo Fisher Scientific, Odense, Denmark) in tandem with an LTQ-Orbitrap Elite mass spectrometer (Thermo Fisher Scientific, Bremen, Germany). MS/MS scans in the range of *m/z* 350 to 1800 were recorded with a mass resolution of 70,000 at *m/z* 400. The LC–MS/MS data were acquired in data-dependent mode, and the ten most intense precursor ions were isolated and fragmented by collision-induced dissociation (CID) with 32% normalized collision energy. Dynamic exclusion was enabled (exclusion list size: 500, exclusion duration: 40 s).

### Database search and bioinformatics

The MS/MS data were searched against the NCBI Sus_refesq_20180716.fasta (63,695 sequences) Fasta database for peptide identification and quantification using Mascot 2.5.1 and Proteome Discoverer 1.4 (Thermo). The search parameters were specified as follows: one missed enzymatic cleavage site was allowed, the mass tolerance was set to 10 ppm for precursor ions and ± 0.05 Da for fragment ions, carbamidomethylation was set as the fixed modification, and oxidation and iTRAQ-4plex were set the variable modifications. The false-positive detection rate (FDR) was calculated using a decoy database search with a FDR < 1.0%, which allowed each protein to be identified by at least 1 specific polypeptide, normalized to the median of the data. We compared the expression levels of all the identified proteins between the TP and YP groups to identify the proteins involved in reproductive traits of boars on plateaus. Student’s t-test was used to compare differences in protein expression between the TP and YP groups and to calculate the p values. *p* < 0.05 and a fold change ≥ 1.5 or ≤ 0.67 were set as the thresholds to identify differentially expressed proteins (DEPs). The average of six labeled sample mixtures was used as a reference (ref) based on the weighted average of the intensity of the reported ions for each identified peptide. The final ratios of proteins were normalized according to the median average protein ratio for the mixtures of different labeled samples (TP1/ref, TP2/ref, YP1/ref, and YP2/ref).

The DEP data were analyzed using bioinformatics, and the UniProt IDs of the DEPs were converted into mouse UniProt IDs due to the small number of studies on gene function in pigs. The gene ontology (GO) annotation and DEP enrichment were analyzed using the GO consortium database for GO assignment (http://geneontology.org/). Kyoto Encyclopedia of Genes and Genomes (KEGG) pathway and protein–protein interaction (PPI) analyses were performed using STRING online software (https://string-db.org/). The results of the GO analysis were mapped into a senior bubble map using the OmicShare tool, a free online platform for data analysis (http://www.omicshare.com/tools), which was also used to map the volcano figure and heatmap. The PPI networks were visualized and analyzed using Cytoscape 3.2.1 software [13].

### Validation of DEPs by western blot analysis

From the differentially expressed proteins, we randomly selected four proteins, Cofilin-1 (CFL1), pro-epidermal growth factor (EGF), fibronectin 1 (FN1), and glutathione peroxidase 4 (GPX4), for western blot analysis (three replicates) to validate their expression levels in TP and YP spermatozoa; beta actin (β-actin) was used as a loading control. The bar line charts were created using Sigmaplot 10.0 (Systat Software, San Jose, CA, USA). In brief, denatured sperm proteins (30 µg) from TP and YP were separated by sodium dodecyl sulfate–polyacrylamide gel electrophoresis (SDS-PAGE with a 4% stacking gel and 12% separating gel) and transferred to polyvinylidene fluoride (PVDF) membranes using a Hoefer TE22 blotting instrument (Hoefer, Holliston, MA, USA). The membranes were blocked overnight in blocking buffer (P0071, Shanghai Beyotime Biotechnology Co. Ltd, Shanghai, China), incubated with the appropriate primary antibody (1:1000, ab42824, ab231103, ab32419, ab231174 or ab8227, Abcam, Cambridge, UK) and gently shaken at room temperature for 2 h. After three washes with phosphate-buffered saline containing 0.1% Tween 20 (PBST), the membranes were incubated with the appropriate secondary antibody (1:1000, A0208, Beyotime Ltd., Shanghai, China) for 1 h. After three washes in Tris-buffered saline with Tween 20 for 30 min, the immune complexes on the membranes were visualized using BeyoECL Plus (P0018S, A0216, Beyotime Ltd., Shanghai, China) following the manufacturer’s instructions. To determine the expression levels of CFL1, EGF, FN1 and GPX4 relative to that of β-actin, the gray value of the bands was analyzed using ImageJ 1.44 (NIH, Bethesda, MA, USA).

### Statistical analysis

Statistical analyses were performed using IBM SPSS Statistics v17.0 (SPSS, Inc., Released 2008. SPSS Statistics for Windows, Version 17.0. Chicago: SPSS, Inc.). Graphs were prepared using SigmaPlot 10.0 (Systat Software, San Jose, CA, USA). Homogeneity of variance and one-way analysis of variance (ANOVA) were used to determine the significance of differences between two groups. All quantitative data are presented as the mean ± standard deviation (S.D.). We considered *P* < 0.05 (*) as statistically significant and *P* < 0.01 (**) as extremely statistically significant.

## Results

### Comparison of sperm quality in the TP and the YP

The results showed that the hypothesis of homogeneity variance was satisfactory (sig > 0.05), and the sperm concentration and motility were statistically higher but abnormalities were statistically lower in TP than in YP, although there were no significant differences in the average path velocity (VAP), curvilinear velocity (VCL), straight line velocity (VSL) or ROS fluorescence unit (RFU, which represents the level of intracellular ROS) of spermatozoa between the and YP (Table 1). Obviously, the sperm quality of TP was superior to that of YP on plateaus.

**Table 1:** Sperm quality analysis of TP and YP

Semen	Concentration (10^8^/mL)	Motility (%)	VAP (µm/s)	VCL (µm/s)	VSL (µm/s)	Abnormality (%)	RFU
TP	3.81 ± 0.22**	86.00 ± 1.63**	21.59 ± 0.52	46.17 ± 6.80	18.37 ± 3.82	8.61 ± 0.24*	17.73 ± 3.81
YP	2.91 ± 0.13	73.00 ± 3.00	20.03 ± 0.54	43.07 ± 6.20	17.37 ± 3.29	9.40 ± 0.17	25.40 ± 4.42

^*^ Note: Each bar represents the mean ± standard deviation (SD); * Significant difference (*p* < 0.05), **Highly significant difference (*p* < 0.01). VAP = Average path velocity; VCL = Curvilinear velocity; VSL = Straight line velocity; RFU = Reactive oxygen species (ROS) fluorescence unit, which represent the sperm ROS level; TP = Tibetan pig (n = 10); YP = Yorkshire pig (n = 10)

### DEP identification

**Fig. 1:**
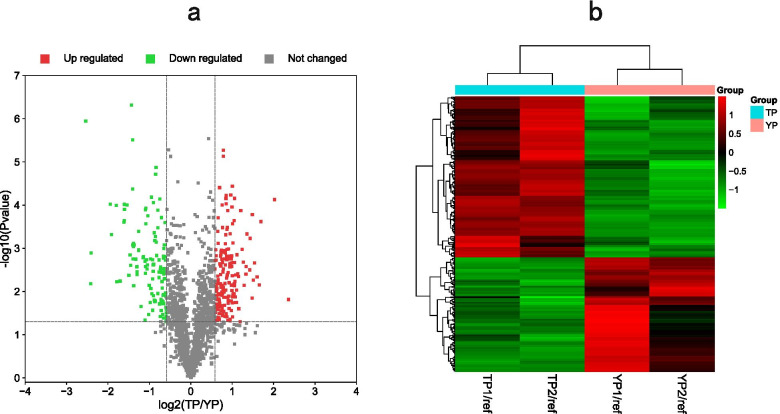
Comparison of the differentially expressed proteins (DEPs) from four labeled samples. **(a)** Volcano plots of the DEPs. Each point in the volcanic map represents a protein; the abscissa represents the logarithmic value of the expression of a certain protein in the two samples. The red points represent upregulated DEPs, the green points represent downregulated DEPs, and the black dots represent nondifferentially expressed proteins. **(b)** Hierarchical clustering heat maps of the DEPs. The expression of each protein is illustrated in red and green to indicate high and low expression, respectively. TP = Tibetan pig (*n* = 10); YP = Yorkshire pig (*n* = 10)

Overall, 33,093 spectra were obtained from the LC–MS/MS analysis. A total of 1,555 proteins were detected from 6,375 unique peptides by quantitative proteomic analysis (Additional file 1: Table S1). Among the proteins detected, 318 DEPs were detected from two biological duplicates (*P* < 0.05 and a fold change ≥ 1.5 or ≤ 0.67). Of the DEPs identified, 186 proteins were upregulated and 132 proteins were downregulated in TP compared with those in YP (the control group; Fig. 1a and additional file 2: Table S2). Cluster analysis based on the protein abundance data of the 318 DEPs showed that the two biological duplications in each breed were clustered into one group and that TP1, TP2, YP1 and YP2 were clustered together (Fig. 1b and Additional file 3: Table S3).

### GO and KEGG enrichment analysis of DEPs

**Fig. 2:**
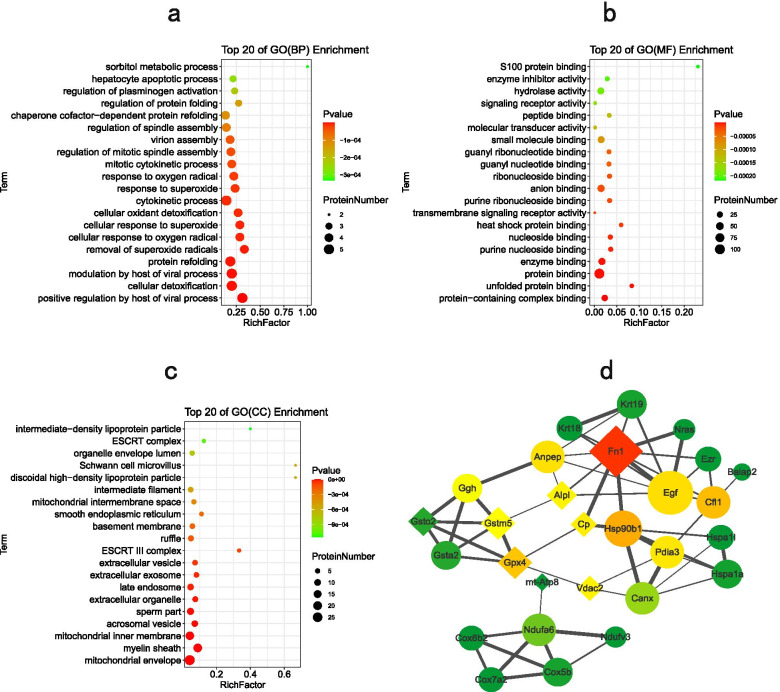
Gene ontology (GO) enrichment analysis and protein–protein interaction (PPI) network construction. **(a)** The top 20 GO (biological process, BP) enrichment terms of the differentially expressed proteins (DEPs); **(b)** The top 20 GO (molecular function, MF) enrichment terms of the DEPs; **(c)** The top 20 GO (cellular component, CC) enrichment terms of the DEPs; **(d)** PPI network of the DEPs involved in important pathways. The nodes represent DEPs, and the edges between the nodes indicate interactions between two connecting DEPs. The node colors indicate the betweenness of the node interaction: the more red the color, the larger the betweenness, indicating a stronger influence in the network. The node sizes indicate the degree of interaction between nodes: the larger the size, the higher the degree, indicating stronger stability in the network. The node shapes represent upregulated proteins (ellipse) or downregulated proteins (diamond). The degrees of edge width represent the protein–protein interaction scores

The DEPs were classified by gene ontology annotation based on three categories: biological process (BP), molecular function (MF), and cellular component (CC) (Additional file 4: Table S4, only result for FDR *P* < 0.05 is displayed). Of the BP terms, sorbitol metabolic process, removal of superoxide radical, cellular response to superoxide, cellular response to oxygen radicals, regulation of protein folding, response to superoxide and regulation of mitotic spindle assembly were enriched in DEPs (Fig. 2a). Of the MF terms, S-100 protein binding, unfolded protein binding, heat shock protein binding, nucleoside binding, ribonucleoside binding, and signaling receptor activity were enriched in DEPs (Fig. 2b). Of the CC terms, mitochondrial envelope, mitochondrial inner membrane, acrosomal vesicle, and sperm part were enriched in DEPs (Fig. 2c).

To determine the potential pathways of the DEPs, KEGG pathway analysis was performed. Forty-one of 318 DEPs were successfully mapped to 13 term IDs (false discovery rate < 0.05, Additional file 5: Table S5), and some important pathways influencing the sperm quality of boars on plateaus are shown in Table 2. These important pathways were mainly regulation of actin cytoskeleton, glutathione metabolism, oxidative phosphorylation, and estrogen signaling pathway, among others.

**Table 2:** Important Kyoto Encyclopedia of Genes and Genomes (KEGG) pathways

Term ID	Term description	Protein counts	Matching proteins in the network (labels)
mmu04216	Ferroptosis	5	Acsl6, Cp, Gpx4, Ica, Vdac2
mmu04810	Regulation of actin cytoskeleton	9	Arpc5l,Baiap2, Cfl1, Egf, Ezr, Fn1, Nras, Rras, Scin
mmu00480	Glutathione metabolism	5	Anpep, Gpx4, Gsta2, Gstm5, Gsto2
mmu04612	Antigen processing and presentation	5	Canx, Hspa1a, Hspa1l, Lgmn, Pdia3
mmu00190	Oxidative phosphorylation	6	Cox5b, Cox6b2, Cox7a2, Ndufa6, Ndufv3, mt-Atp8
mmu00790	Folate biosynthesis	3	Alpl, Cbr1, Ggh
mmu04714	Thermogenesis	8	Acsl6, Cox5b, Cox6b2, Cox7a2, Ndufa6, Ndufv3, Nras, mt-Atp8
mmu04915	Estrogen signaling pathway	6	Hsp90b1, Hspa1a, Hspa1l, Krt18, Krt19, Nras

#### Protein–protein interaction (PPI) network construction and analysis

To further identify candidate proteins among the DEPs involved in important pathways, we constructed a PPI network using STRING and Cytoscape 3.2.1 software (Additional file 6: Table S6—PPI relationships and scores). The PPI network consisted of 28 nodes and 57 edges (Fig. 2d). The details are presented in Additional file 7: Table S7. Based on the protein–protein interaction (PPI) network analysis, we identified 8 candidate proteins (FN1, EGF, HSP90B1, CFL1, GPX4, NDUFA6, VDAC2, CP) that might affect the sperm quality of boars on plateaus (Table 3).

**Table 3:** Candidate proteins that affect the sperm quality of boars on plateaus

Protein description	Accession No	Symbol	The number of matched polypeptides	MW [kDa]	Fold Change*	Regulation*
Fibronectin	XP_003133690.2	FN1	51	289	0.55	Down
Pro-epidermal growth factor precursor	NP_999185.1 (+ 2)	EGF	10	134	1.7	Up
Endoplasmin precursor	NP_999268.1 (+ 1)	HSP90B1	13	93	1.66	Up
Cofilin-1	NP_001004043.1	CFL1	3	19	1.71	Up
Phospholipid hydroperoxide glutathione peroxidase precursor	NP_999572.1	GPX4	10	22	0.47	Down
NADH dehydrogenase [ubiquinone] 1 alpha subcomplex subunit 6	NP_001172107.1	NDUFA6	1	15	1.66	Up
Voltage-dependent anion-selective channel protein 2	NP_999534.1 (+ 1)	VDAC2	11	32	0.37	Down
Ceruloplasmin precursor	NP_001254623.2 (+ 2)	CP	6	122	0.38	Down

**Fig. 3:**
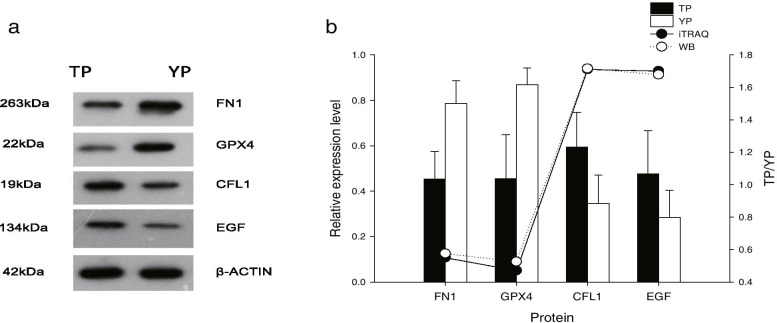
Western blot validation. **(a)** Western blot (WB) analysis of four proteins (CFL1, CYCS, FN1, and GPX4). β-actin was used as a loading control. **(b)** Statistical results of quantitative comparison of the isobaric tag for relative and absolute quantification (iTRAQ) and WB for the four proteins. The bar chart shows the statistical results of WB. The line chart represents the quantitative comparison of iTRAQ and WB for the four proteins. The data for the WB expression levels are represented as the means ± standard deviation (SD); * Significant difference (*P* < 0.05), ** extremely significant difference (*P* < 0.01). TP = Tibetan pig (*n* = 10); YP = Yorkshire pig (*n* = 10)

The results showed that the hypothesis of homogeneity variance was satisfactory (sig > 0.05), and the results of the western blot analysis of the four proteins are presented in Fig. 3a. The gray value of each lane in the western blot was consistent with the iTRAQ quantification level (Fig. 3b).

## Discussion

Previous studies have shown that a significant percentage of reproductive failure is attributed to semen quality [14]. Oxidative stress has been established as one of the main causes of male infertility. Oxidative stress results from high concentrations of free radicals and suppression of the antioxidant potential, which may alter protein expression in spermatozoa [15]. The positive effects of Astragalus polysaccharide on the sperm quality of boars were mainly due to the elimination of excessive mitochondrial ROS, improvement of antioxidant capacity and enhancement of ATP level [16]. Feeding a diet with 500 mg/kg oregano essential oil may result in a decrease in ROS in sperm [17]. Our results also showed that the ROS level of TP spermatozoa was lower than that of YP spermatozoa, although there were no significant differences between the two groups. Sperm concentration and motility were statistically higher but abnormalities were statistically lower in TP than in YP, although the sperm motility parameters average path velocity (VAP), curvilinear velocity (VCL) and straight line velocity (VSL) were not significantly different between the two groups. Obviously, the sperm quality of TP was superior to that of YP on plateaus. In our previous studies, we found that although the sperm VAP of TP was significantly lower in high-altitude plateau areas than that of low-altitude pigs, their sperm counts, motility and abnormalities were equivalent [3]. These results suggested that the male reproductive system of TP was more adaptive to high-altitude conditions than that other migrated pig breeds, such as YP, which provides a good model for studying the regulatory mechanisms of the sperm quality of boars on plateaus.

BP, which has particular functions, has been identified in previous studies using proteomic analyses of spermatozoa in animals. For example, abundant DEPs between Meishan and Duroc boar spermatozoa were found to be primarily involved in energy metabolism, sperm motility, capacitation and sperm-oocyte binding [18]. The DEPs of TP spermatozoa from high and low altitudes were predominantly associated with the actin cytoskeleton, the tricarboxylic acid (TCA) cycle, and adenosine triphosphate (ATP) metabolism [3]. Most of the DEPs from buffalo spermatozoa before and after capacitation were involved in transport, cytoskeleton organization, sexual reproduction, and spermatogenesis [19]. In the present study, we found that the DEPs were predominantly associated with sorbitol metabolic process, removal of superoxide radicals, cellular response to superoxide, response to superoxide and mitotic spindle assembly regulation, among others. These results differ somewhat from our previous research findings, potentially due to the differences in species and environment, and the results of the present study provide new insights into the sperm quality of boars on plateaus.

Sperm quality is closely associated with many KEGG pathways. Previous research has indicated that the DEPs of TP spermatozoa from high and low altitudes were significantly enriched in the pathways: the tricarboxylic acid (TCA) cycle and respiratory electron transport, plate activation, signaling and aggregation, complement and coagulation cascades, vesicle-mediated transport and the actin cytoskeleton [3]. The DEPs from buffalo spermatozoa before and after capacitation were mainly involved in metabolic pathways, the PPAR signaling pathway, and oxidative phosphorylation [19]. In the present study, we found that the DEPs were mainly enriched in the regulation of actin cytoskeleton, glutathione metabolism, oxidative phosphorylation, and estrogen signaling pathway. Among these pathways, glutathione metabolism and oxidative phosphorylation are involved in energy metabolism and amino acid metabolism, respectively, and the identified proteins in the network (labels) included GPX4, NDUFA6, NDUFV3, mt-ATP8, etc. The proteins involved in the actin cytoskeleton regulation and estrogen signaling pathways in the network (labels) included CFL1, EGF, FN1, HSP90B1, etc.

Previous research has indicated that BAG6 and HIST1H2BA are potential candidates as male infertility biomarkers [20]. CMTM4 is associated with spermatogenesis and sperm quality [21]. Sperm mitochondrial dysfunction and oxidative stress are possible causes of isolated asthenozoospermia [22]. The regionally distinct expression and localization of CETN1 and CSPP1 are strongly related to spermiogenesis and sperm morphology maintenance. Obesity is associated with declines in the abundances of CETN1 and CSPP1 and affect sperm morphology in mice and relevant clinical samples. The correlation between altered protein expression in mice and humans suggests that these effects may contribute to poor sperm quality, including increasing the frequency of deformities [23]. Both increased and relaxed sperm competition can have a pronounced impact on the molecular composition of the male gamete [24]. Interestingly, enzymes that are essential in glycolysis/gluconeogenesis, such as HK1, ALDH2, LDHA and LDHC, are markedly upregulated in Meishan spermatozoa compared with those in Duroc spermatozoa [18]. The sperm proteins that are expressed in greater abundance in high- compared with low-fertility bulls are HSP90, ZFP34, IFNRF4, BCL62, NADHD, TUBB3 and histone H1 [25]. In a previous study, enolase-1 (ENO1) was found to be overexpressed in the high-fertility group and Binder of SPerm-1(BSP1) was found to be overexpressed in the low-fertility group [26]. The sperm triosephosphate isomerase (TPI) content and amount of epididymal secretory glutathione peroxidase (GPX5) in seminal plasma may be used as quality markers of boar sperm [27]. ATP citrate lyase is overexpressed in liquid-stored sperm, while cytosolic nonspecific dipeptidase is overexpressed in fresh boar sperm samples [28]. These results differ somewhat from our research findings, potentially due to differences in the species and environment.

In this study, based on GO, KEGG enrichment and PPI network analyses, we identified 8 candidate proteins (FN1, EGF, HSP90B1, CFL1, GPX4, NDUFA6, VDAC2, CP) that may affect the sperm quality of boar on plateaus. Among the 8 candidate proteins identified, we found that EGF, HSP90B1, CFL1, and NDUFA6 were upregulated in TP spermatozoa and were enriched in the regulation of the actin cytoskeleton, glutathione metabolism, oxidative phosphorylation, and the estrogen signaling pathway(Table 2). Research has shown that EGF promotes the proliferation and differentiation of mouse spermatogenic cells [29]. In a previous study, HSP90B1 was found to be involved in protein folding and in the targeting of misfolded proteins for endoplasmic reticulum-associated degradation; additionally, it participated in calcium storage, which is required for the normal functions of spermatozoa [30], and was shown to be upregulated in the normozoospermic group but downregulated in the asthenozoospermic group in comparison to that in the control group [31]. PKA-dependent phosphorylation of LIMK1 and cofilin is essential for mouse sperm acrosomal exocytosis [32]. NDUFA6 of mitochondrial complex I anchors an acyl carrier protein and is essential for catalytic activity [33]; downregulation of the NDUFA6 subunit has been linked to the inactivation of complex I, leading to the induction of apoptosis [34]. Consequently, sperm quality is promoted by the expression of four proteins (EGF, HSP90B1, CFL1 and NDUFA6) that were found to be upregulated in TP spermatozoa. Among the 8 candidate proteins, we found that the expression levels of four proteins (FN1, GPX4, VDAC2 and CP) were downregulated in TP spermatozoa and were enriched in the regulation of the actin cytoskeleton, ferroptosis, and glutathione metabolism (Table 2). Previous studies have shown that fibronectin 1 (FN1) is downregulated in the spermatozoa of Tibetan pigs living at high versus low altitudes [3]. The level of FN1 in fresh seminal plasma from boar semen may be used as a sperm freezability marker, facilitating the use of frozen-thawed boar spermatozoa [35]. Glutathione peroxidase 4 (GPX4) is essential for spermatogenesis; heterozygous expression of a catalytically inactive mutant form of Gpx4 impairs spermatogenesis, and well-balanced expression of functional GPX4 has emerged as a prerequisite for complete male fertility [36]. Mitochondrial GPX4 forms the mitochondrial sheath of spermatozoa and thus guarantees male fertility [37]. Downregulation of VDAC2 inhibits spermatogenesis via the JNK/P53 cascade [38]. In a previous study, the fertility-related protein markers ENO1, ATP5B, VDAC2, GPX4, and UQCRC2 were identified in 20 individual bull semen samples [39]. CP is a Cu-containing protein and an oxidase with a high antioxidation capacity that is involved in scavenging oxygen free radicals and protecting various organs from lipid peroxidation and other types of oxidative attack in the extracellular space [40]. Dietary addition of Mo indirectly increases oxidative stress, exacerbating Cd toxicity by reducing the expression of MT and CP, which protect testicles from lipoperoxidation and other types of oxidative stress [41]. Thus, the downregulated expression of the four proteins (FN1, GPX4, VDAC2 and CP) in TP spermatozoa supports sperm quality.

## Conclusions

In summary, the sperm quality of TP is better than that of YP on plateaus, which indicates that the male reproductive system of TP is more adaptive to high-altitude conditions than that of YP. The proteins identified by comparative proteomic analyses of spermatozoa between TP and YP raised at high altitudes were mainly enriched in the regulation of the actin cytoskeleton, glutathione metabolism, oxidative phosphorylation, and the estrogen signaling pathways. We found 8 candidate proteins (FN1, EGF, HSP90B1, CFL1, GPX4, NDUFA6, VDAC2, CP) that might affect the sperm quality of boars on plateaus. These proteins may be candidate proteins for assessing the sperm quality of boars on plateaus and may provide a reference for further studies on improving sperm quality and the molecular breeding of boars on plateaus.

## Supplementary Information

**Additional file 1: Table S1.** A list of all identified proteins.

**Additional file 2: Table S2.** A list of the volcano plots.

**Additional file 3: Table S3.** A list of the heatmap.

**Additional file 4: Table S4.** Go analysis of DEPs.

**Additional file 5: Table S5.** KEGG analysis of DEPs.

**Additional file 6: Table S6.** The PPI relationships and scores.

**Additional file 7: Table S7.** The details of PPI network.

## Data Availability

All data generated or analyzed during this study are included in this published article and its supplementary information files.

## Abbreviations

HT: High-altitude
LT: Low-altitude
CASA: Computer-assisted semen analysis
VAP: Average path velocity
LC–MS/MS: Liquid chromatography-tandem mass spectrometry
iTRAQ: Isobaric tag for relative and absolute quantification
FASP: Filter-assisted sample preparation
DEPs: Differentially expressed proteins
GO: Gene ontology
BP: Biological process
MF: Molecular function
CC: Cellular component
KEGG: Kyoto Encyclopedia of Genes and Genomes
PPI: Protein–protein interaction
PVDF: Polyvinylidene fluoride
SDS-PAGE: Sodium dodecyl sulfate–polyacrylamide gel electrophoresis
